# HSPA13 facilitates NF-κB–mediated transcription and attenuates cell death responses in TNFα signaling

**DOI:** 10.1126/sciadv.abh1756

**Published:** 2021-10-06

**Authors:** Chun Gao, Jianhua Deng, Hanchenxi Zhang, Xinran Li, Shuchen Gu, Mingjie Zheng, Mei Tang, Yezhang Zhu, Xin Lin, Jianping Jin, Long Zhang, Jun Huang, Jian Zou, Zong-Ping Xia, Ping-Long Xu, Li Shen, Bin Zhao, Xin-Hua Feng

**Affiliations:** 1 The MOE Key Laboratory of Biosystems Homeostasis and Protection and Innovation Center for Cell Signaling Network, Life Sciences Institute, Zhejiang University, Hangzhou, Zhejiang 310058, China.; 2 Zhejiang Provincial Key Laboratory of Cancer Molecular Cell Biology, Life Sciences Institute, Zhejiang University, Hangzhou, Zhejiang 310058, China.; 3 Cancer Center, Zhejiang University, Hangzhou, Zhejiang 310058, China.; 4 Eye Center of the Second Affiliated Hospital School of Medicine, Institutes of Translational Medicine, Zhejiang University, Hangzhou, Zhejiang 310058, China.; 5 Institute for Immunology, Tsinghua University School of Medicine, Tsinghua University–Peking University Jointed Center for Life Sciences, Beijing 100084, China.; 6 The First Affiliated Hospital, Zhengzhou University, Zhengzhou, Henan 450052, China.; 7 The Second Affiliated Hospital, Zhejiang University, Hangzhou, Zhejiang 310058, China.

## Abstract

RIP1 has emerged as a master regulator in TNFα signaling that controls two distinct cellular fates: cell survival versus programmed cell death. Because the default response of most cells to TNFα is NF-κB–mediated inflammation and survival, a specific mechanism must exist to control the divergence of signaling outcome. Here, we identify HSPA13 as a transcription-independent checkpoint to modulate the role of RIP1 in TNFα signaling. Through specific binding to TNFR1 and RIP1, HSPA13 enhances TNFα-induced recruitment of RIP1 to TNFR1, and consequently promotes downstream NF-κB transcriptional responses. Meanwhile, HSPA13 attenuates the participation of RIP1 in cytosolic complex II and prevents cells from programmed death. Loss of HSPA13 shifts the transition of RIP1 from complex I to complex II and promotes both apoptosis and necroptosis. Thus, our study provides compelling evidence for the cellular protective function of HSPA13 in fine-tuning TNFα responses.

## Teaser

HSPA13 is a novel checkpoint regulator in the TNFα signaling pathway controlling cellular physiological outcomes.

## INTRODUCTION

Tumor necrosis factor-α (TNFα) is a pleiotropic cytokine that ignites various cellular outcomes in different cell types such as cell survival, proliferation, differentiation, induction of antimicrobial activity, and programmed cell death including apoptosis and necroptosis (*1*, *2*). Dysregulation of TNFα signaling has been implicated in many human diseases such as neurodegeneration, inflammation, autoimmune diseases, and cancer (*3*).

Receptor-interacting protein 1 (RIP1) is a key regulator situating at the crossroad of TNFα signaling network that strategically controls multiple downstream signaling pathways with distinct outcomes (*4*). Binding of TNFα to TNF receptor 1 (TNFR1) results in rapid trimerization of TNFR1 and assembly of the membrane-associated complex I that contains TNFα-bound TNFR1, TNF receptor–associated death domain protein (TRADD), RIP1, and TNF receptor–associated factor 2 (TRAF2) (*5*–*8*). TRAF2, in turn, recruits cellular inhibitor of apoptosis protein 1 (cIAP1) and cIAP2 and stabilizes them by preventing their auto-ubiquitination (*9*, *10*). In complex I, RIP1 is K63-polyubiquitinated on K377 by cIAP1 and cIAP2, generating a docking site for the IκB kinase (IKK) complex and the transforming growth factor–β–activated kinase-1 (TAK1) complex, which subsequently drives the activation of prosurvival nuclear factor κB (NF-κB) signaling (*11*, *12*).

Depending on the cellular context, long-term TNFα stimulation leads to RIP1 dissociation from complex I and its deubiquitination by deubiquitinase CYLD or A20 (*13*–*15*). Once RIP1 is released, it is primed to initiate assembly of a cytosolic death-initiating signaling complex (DISC) by Fas–associated death domain protein (FADD) and procaspase 8, also referred to as complex IIa (*16*, *17*). Caspase 8 is then processed from its procaspase form to activate apoptosis in a RIP1 kinase–dependent manner (*16*). When caspase 8 activation fails or is inhibited, complex IIa evolves to form complex IIb, also known as necrosome, which consists of RIP1, RIP3, and mixed-lineage kinase domain–like pseudokinase (MLKL) (*18*–*23*). This RIP1-mediated necroptosis requires the kinase activity of RIP1 (*18*), which activates RIP3 and then transmits death signaling to the necroptosis executioner MLKL.

Although the precise mechanism as to how complex I converts into complex II is poorly understood, it is clear that deubiquitination of RIP1 and its dissociation from TNFR1 are two key events that switch complex I–initiated inflammatory response to complex IIa/b–initiated cell death. However, the fact that most cell types do not succumb to TNFα stimuli indicates that complex II is actively suppressed in cells (*24*). In agreement, recent studies have revealed the in vivo role of RIP1 as an essential survival factor by suppressing both caspase 8–dependent apoptosis and RIP3/MLKL-dependent necroptosis (*25*–*28*). These findings strongly suggest the existence of specific checkpoints that effectively bifurcate survival versus death signaling. Therefore, understanding the dynamics of RIP1 activity is important in elucidating the complexity and functions of TNFα signaling network.

Heat shock 70-kDa protein 13 (HSPA13) is a member of HSP70 family that also encodes a conserved adenosine triphosphatase (ATPase) domain (*29*). Current knowledge regarding the biological function of HSPA13 is limited. Here, we identify HSPA13 as a previously unidentified regulator of RIP1. Our finding that HSPA13 interacts with both RIP1 and TNFR1 suggests the presence of a TNFR1-HSPA13-RIP1 complex, with HSPA13 being the bridging factor. These protein-protein interactions sequester RIP1 in complex I and prevent its transition into cytoplasmic complex IIa or complex IIb, thereby controlling the outcome of TNFα signaling.

## RESULTS

### HSPA13 is an interacting partner of RIP1

**Fig. 1.**
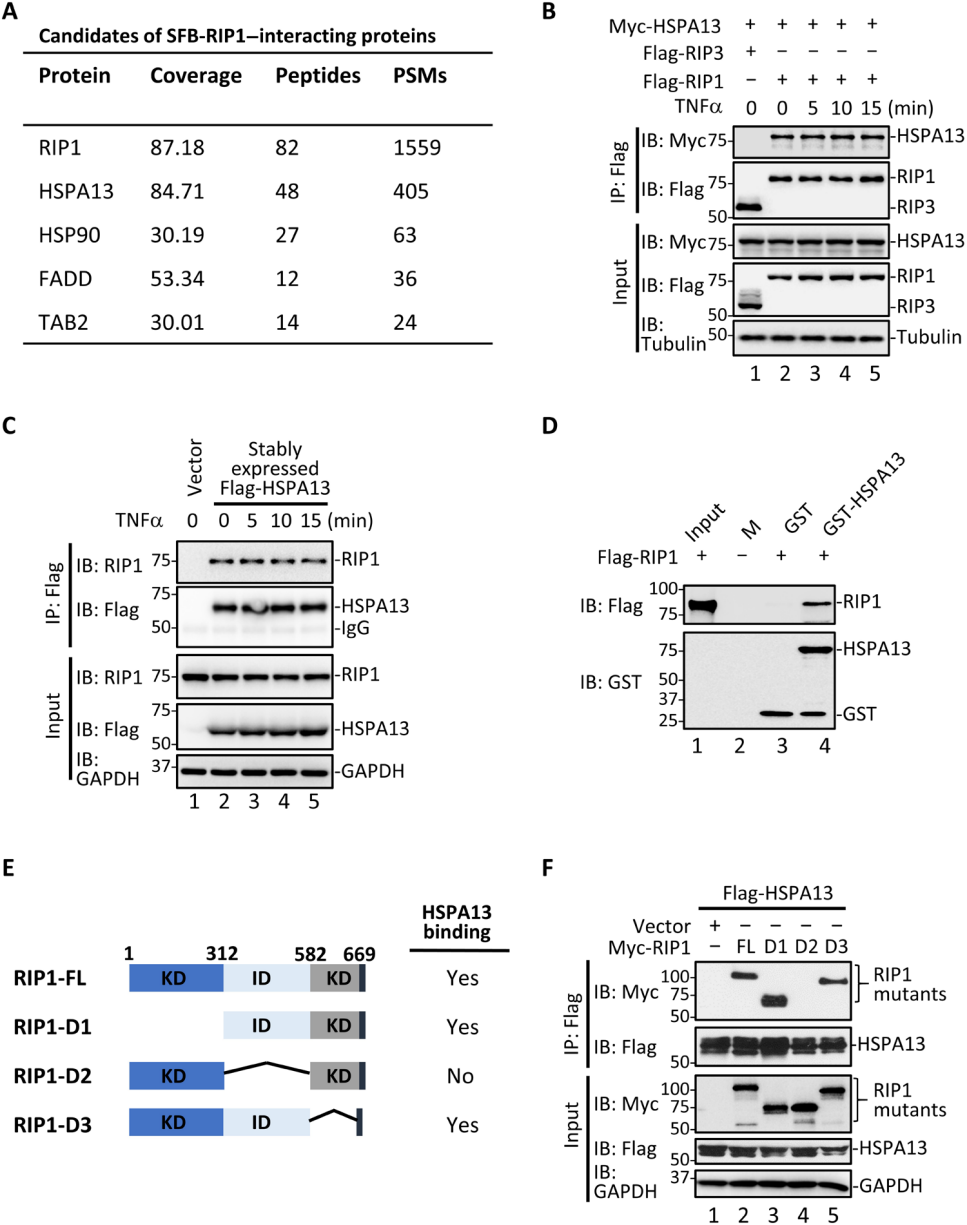
HSPA13 specifically interacts with RIP1. (**A**) HEK293T cells were transfected with SFB-tagged RIP1. RIP1-containing complexes were purified and subsequently analyzed by mass spectrometry. PSMs, peptide-spectrum matches. (**B**) Flag-RIP1 or Flag-RIP3 was cotransfected with Myc-HSPA13 into HEK293T cells. After 24 hours, cells were treated with TNFα (10 ng/ml) for the indicated time. Same concentration of TNFα was used unless noted. Co-IP was carried out with anti-Flag. IP, immunoprecipitation; IB, immunoblotting. (**C**) HT29 cells stably expressing Flag-HSPA13 were stimulated with TNFα for the indicated time. Co-IP was carried out with anti-Flag. GAPDH, glyceraldehyde-3-phosphate dehydrogenase. (**D**) In vitro binding assay was carried out with purified recombinant GST-tagged HSPA13 and in vitro translated RIP1. M, protein marker. (**E**) Diagram of RIP1 deletion mutants used in domain-mapping experiments. KD, kinase domain; ID, intermediate domain; DD, death domain. (**F**) Myc-RIP1 mutants and Flag-HSPA13 were cotransfected into HEK293T cells. After 24 hours, co-IP was carried out with anti-Flag.

To characterize the role of RIP1 in controlling TNFα signaling, we isolated the RIP1 interactome using human embryonic kidney (HEK) 293T cells that transiently expressed N-terminally SFB-tagged (S-protein tag, Flag epitope tag, and streptavidin-binding peptide tag) RIP1 (SFB-RIP1). Mass spectrometry analysis revealed a number of previously reported RIP1-associated proteins, including the heat shock protein 90-α (HSP90) (*30*), FADD (*31*), and TAK1-binding protein 2 (TAB2) (*32*). Among these, HSPA13 had the highest score with 84.71% protein peptide sequence coverage and high peptide-spectrum matches (PSMs), suggesting that it is a promising candidate as an interacting partner of RIP1 (Fig. 1A).

We validated the HSPA13-RIP1 interaction both in vivo and in vitro. In HEK293T cells that transiently expressed Myc-HSPA13 and Flag-RIP1, we observed that HSPA13 specifically interacted with RIP1 (Fig. 1B, lanes 2 to 5), but not a related kinase RIP3 (Fig. 1B, lane 1) in a co-immunoprecipitation (co-IP) assay. Without an efficient HSPA13 antibody suitable for IP, we generated an HT29 cell line stably expressing Flag-HSPA13 and used anti-Flag antibody for IP. As shown in Fig. 1C, HSPA13 strongly bound to endogenous RIP1 (lanes 2 to 4). Notably, the HSPA13-RIP1 interaction apparently occurred independent of TNFα stimulation (Fig. 1, B, lanes 2 to 5, and C, lanes 2 to 5). Furthermore, recombinant glutathione *S*-transferase (GST)–HSPA13 could bind to in vitro translated RIP1 protein in an in vitro GST pulldown assay (Fig. 1D), demonstrating a direct interaction between HSPA13 and RIP1. To understand the structural requirements for the interaction between HSPA13 and RIP1, we generated a series of internal deletion mutants of RIP1 and compared their interactions to HSPA13 (Fig. 1E). Co-IP analysis showed that deletion of the intermediate domain of RIP1 completely abolished the interaction between HSPA13 and RIP1 (Fig. 1F, lane 4), suggesting that the intermediate domain of RIP1 mediates its binding to HSPA13.

### HSPA13 is a previously unrecognized component of complex I

**Fig. 2.**
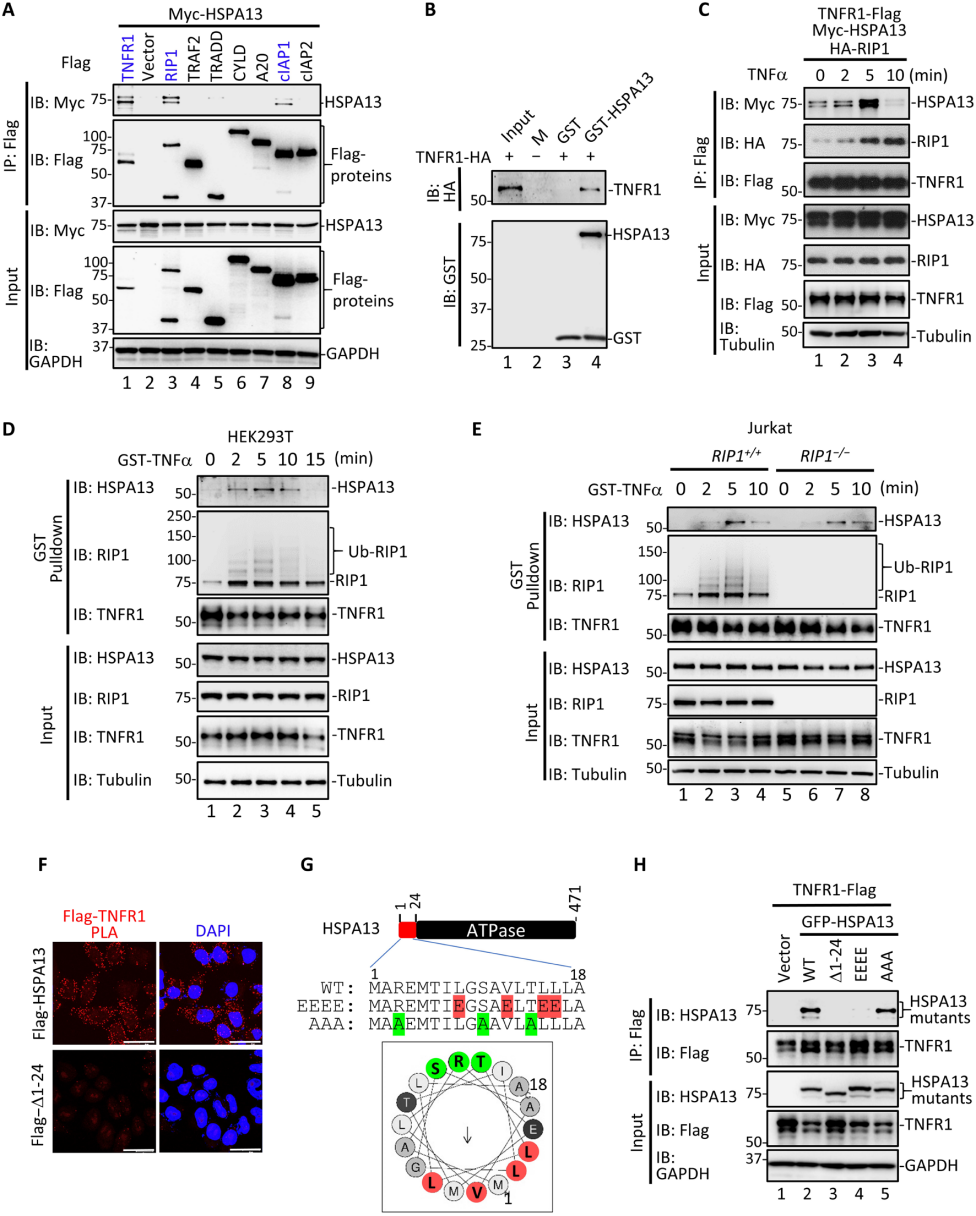
HSPA13 is a previously unidentified component of TNFR1-associated complex I. (**A**) HEK293T cells were transfected with indicated plasmids. After 24 hours, co-IP was carried out with anti-Flag and IP samples were analyzed by Western blotting using the indicated antibody. (**B**) In vitro binding assay was carried out with purified recombinant GST-tagged HSPA13 and in vitro translated TNFR1. (**C**) HEK293T cells were transfected with indicated plasmids. After 24 hours, cells were treated with TNFα for the indicated time. Co-IP was carried out with anti-Flag. (**D**) HEK293T cells were stimulated with GST-TNFα (1 μg/ml) for the indicated time. For *t* = 0 min, GST-TNFα was added at the time immediately after cell lysis. Complex I was purified using glutathione-Sepharose 4B beads. Ub, ubiquitination. (**E**) Jurkat cells were treated with GST-TNFα and precipitation was carried out, as described in (D). (**F**) HeLa cells stably expressing Flag-HSPA13 or Flag–Δ1-24 mutant were labeled with PLA signal (red) and stained with 4′,6-diamidino-2-phenylindole (DAPI; blue). Images were taken under confocal microscopy. Scale bars, 20 μm. (**G**) Linear and helical wheel projection of the N-terminal motif of HSPA13 (amino acids 1 to 18). The residues on the polar face were labeled in green, and the hydrophobic residues were highlighted in red. EEEE indicates the substitution of four red hydrophobic residues with glutamic acid (E), while AAA indicates the substitution of three green polar residues with alanine (A). (**H**) HEK293T cells were transfected with indicated plasmids. After 24 hours, co-IP was carried out with anti-Flag.

Through its strong physical interaction with RIP1, HSPA13 may play an important role in regulating functions of RIP1. We thus determined whether HSPA13 participated in complex I for survival/inflammation or complex II for cell death. Co-IP analysis showed that HSPA13 strongly interacted with TNFR1 and RIP1, while it bound to cIAP1 weakly (Fig. 2A). In sharp contrast, HSPA13 did not coprecipitate with TRADD, TRAF2, CYLD, A20, and cIAP2. Furthermore, HSPA13 did not interact with caspase 8, c-FLIP, FADD, RIP3, and MLKL, key components in complex IIa/b (fig. S1, A and B). The direct interaction between HSPA13 and TNFR1 was observed in an in vitro GST pulldown assay (Fig. 2B, lane 4). These observations suggest a potential participation of HSPA13 as a component in complex I. Co-IP experiments using overexpressed proteins showed that the interaction between HSPA13 and TNFR1 was induced within 5 min of TNFα stimulation (Fig. 2C, lane 3).

To further purify endogenous complex I, we stimulated cells with GST-TNFα and then isolated the ligand-bound complex I by affinity purification. Notably, TNFα induced transient recruitment of HSPA13 to native complex I in both HEK293T and Jurkat cells (Fig. 2, D, lanes 2 to 4, and E, lanes 3 and 4), and the pattern of HSPA13 recruitment to TNFR1 coincided with that of RIP1. Similar phenomena were observed in HT29 cells stably expressing Flag-HSPA13 (fig. S1C). To exclude the possibility that HSPA13 was brought to TNFR1 by RIP1, we took advantage of the *RIP1*-null Jurkat cell line (*33*). As seen in Fig. 2E, the association between TNFR1 and HSPA13 remained unchanged in the absence of RIP1, indicating that the TNFα-induced recruitment of HSPA13 to complex I is independent of RIP1. On the other hand, we treated cells with TNFα plus Smac mimetic (T/S) to induce RIP1-dependent apoptosis (*16*), and performed IP using an anti–caspase 8 antibody to isolate complex IIa. The result of semi-endogenous co-IP showed that Flag-HSPA13 was not detected in cytosolic complex IIa upon T/S treatment even when RIP1 was strongly recruited to caspase 8 (fig. S1D). Together, these data suggest that HSPA13 is a potential factor in TNFα signaling, which is restricted in the membrane-associated complex I but not recruited into the cytosolic complex IIa.

HSPA13 processes a hydrophobic signal sequence at N-terminal (amino acids 1 to 24), which is predicted to control its membrane-associated localization (fig. S2, A and C) (*29*). We then validated the requirement of HSPA13 membrane-bound distribution for its interaction with RIP1 and TNFR1. ∆1-24 mutant failed to interact with TNFR1 (fig. S2B, lane 3) but retained its ability to associate with RIP1 (fig. S2D). To further visualize the in situ TNFR1-HSPA13 interaction, we performed proximity ligation assays (PLAs) in HeLa cells stably expressing Flag-HSPA13 or Flag–∆1-24. The binding of Flag-HSPA13 to endogenous TNFR1 produced strong PLA fluorescent puncta, which were mostly absent in HSPA13–∆1-24 mutant (Fig. 2F). To identify the binding site of HSPA13 for TNFR1, we did a series of deletion mutation within the N-terminal hydrophobic sequence of HSPA13. Unfortunately, unlike the Δ1-24 mutants, none of these deletion mutations abolished the TNFR1-HSPA13 interaction (fig. S2E). Helical wheel prediction suggests that the residues (amino acids 1 to 18) of HSPA13 might be an amphipathic helix, with one face made of hydrophobic residues (L8, V12, L15, and L16) and the other one containing polar residues (R3, S10, and T14) (Fig. 2G) (*34*). Point mutation on the hydrophobic face (EEEE mutation) abolished the HSPA13-TNFR1 interaction, while the AAA mutation on the polar face did not (Fig. 2H). These observations demonstrated that the interaction between TNFR1 and HSPA13 is dependent on the hydrophobic sequence within HSPA13.

### HSPA13 links RIP1 to TNFR1-associated complex I and modulates its pleiotropic activity

**Fig. 3.**
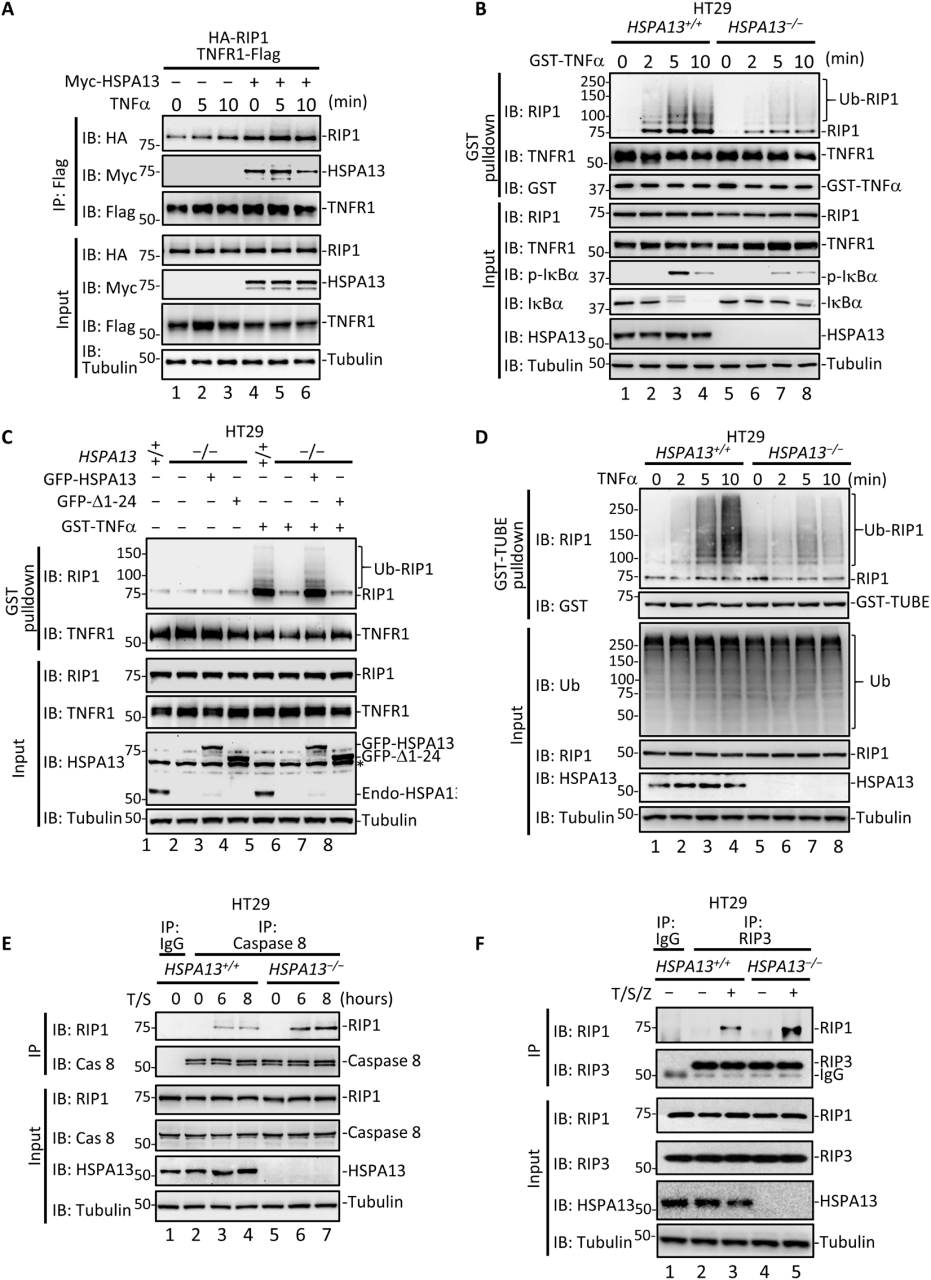
HSPA13 regulates RIP1 signaling complexes. (**A**) HEK293T cells were transfected with indicated plasmids. After 24 hours, cells were treated with TNFα for the indicated time. Co-IP was carried out with anti-Flag. (**B**) HT29 *HSPA13*^−/−^ cell line was generated by using CRISPR-Cas9 system. HT29 parental (*HSPA13^+/+^*) or *HSPA13*^−/−^ cells were stimulated with GST-TNFα for the indicated time. Complex I was purified using glutathione-Sepharose 4B beads. p-IκBα, IκBα-phospho-S32; endo-HSPA13, endogenous HSPA13. (**C**) HT29 parental cells or *HSPA13*^−/−^ HT29 cells that stably expressed GFP, GFP-HSPA13, or GFP–Δ1-24 mutant were treated with GST-TNFα for 5 min. Complex I was purified and analyzed as described in (B). (**D**) HT29 *HSPA13^+/+^* or *HSPA13*^−/−^ cells were treated with TNFα for the indicated time. The ubiquitylated proteins were isolated by GST-TUBE. (**E**) HT29 *HSPA13^+/+^* or *HSPA13*^−/−^ cells were treated with TNFα (20 ng/ml; T) and 50 nM Smac mimetic (S) for the indicated time. Same concentration of T/S was used to induce apoptosis unless noted. Co-IP was carried out with anti–caspase 8 or control immunoglobulin G (IgG) antibody. (**F**) HT29 *HSPA13^+/+^* or *HSPA13*^−/−^ cells were treated with TNFα (20 ng/ml; T), 20 nM Smac mimetic (S), and 20 μM Z-VAD (Z) for 6 hours. Same concentration of T/S/Z was used to induce necroptosis unless noted. Co-IP was carried out with anti-RIP3 or control IgG antibody.

Our finding that HSPA13 is a novel factor in complex I prompted us to investigate the function of HSPA13 in TNFα-induced signaling complex assembly. We found that overexpression of HSPA13 enhanced the TNFR1-RIP1 interaction in response to TNFα (Fig. 3A, compare lane 5 to lane 2). To examine the effect of HSPA13 deficiency on the TNFR1-RIP1 interaction, we generated *HSPA13*-ablated HT29 cells using CRISPR-Cas9 system. Deletion of HSPA13 did not affect the expression level of either TNFR1 or RIP1 (fig. S3, A and B). The recruitment of RIP1 into complex I was markedly decreased upon TNFα treatment in *HSPA13*^−/−^ cells (Fig. 3B, compare lanes 6 to 8 to lanes 2 to 4). To rule out the off-target effects of CRISPR-Cas9–mediated knockout, we stably expressed green fluorescent protein (GFP)–HSPA13 and GFP–∆1-24 in *HSPA13*^−/−^ HT29 cells. The level of the reconstituted HSPA13 was similar to that of the endogenous HSPA13 (fig. S3C). Notably, reconstitution of GFP-HSPA13 fully rescued the TNFα-induced recruitment of RIP1 into native complex I (Fig. 3C, lane 7). In sharp contrast, the Δ1-24 mutant with defect in TNFR1 binding failed to restore the complex I assembly (Fig. 3C, lane 8). All these data suggest that HSPA13 is a scaffolding factor that stabilizes the complex between TNFR1 and RIP1.

Because the engagement of RIP1 in complex I is necessary for its ubiquitination by cIAP1/2, it is convincing that HSPA13 deficiency resulted in decline in TNFα-induced ubiquitination of RIP1 (Fig. 3, B and C). The effect of HSPA13 in mediating RIP1 ubiquitination was further confirmed using tandem ubiquitin binding entity (TUBE) assay (Fig. 3D, compare lanes 7 and 8 to lanes 3 and 4). Consistently, TNFα-induced K63 ubiquitination of RIP1 was notably induced by HSPA13 overexpression (fig. S4).

As RIP1 ubiquitination is critical for complex I to complex II transition, we next examined whether HSPA13 could modulate the formation of complex IIa/IIb. Knockout of *HSPA13* enabled a stronger interaction of RIP1 with caspase 8 upon T/S treatment (Fig. 3E, compare lane 7 to lane 4), suggesting a substantial increase in the formation of complex IIa. In a parallel experiment to initiate necroptosis, we treated HT29 cells with TNFα combined with Smac mimetic and pan-caspase inhibitor Z-VAD-fmk (T/S/Z) and then performed analysis of complex IIb/necrosome by IP using an anti-RIP3 antibody. We found that HSPA13 deficiency could also apparently facilitate formation of complex IIb (Fig. 3F, compare lane 5 to lane 3). These data indicate that HSPA13 facilitates the assembly of RIP1 into complex I and simultaneously prevents RIP1 integration into the cytosolic death complexes IIa/IIb.

### HSPA13 facilitates TNFα-induced NF-κB response

**Fig. 4.**
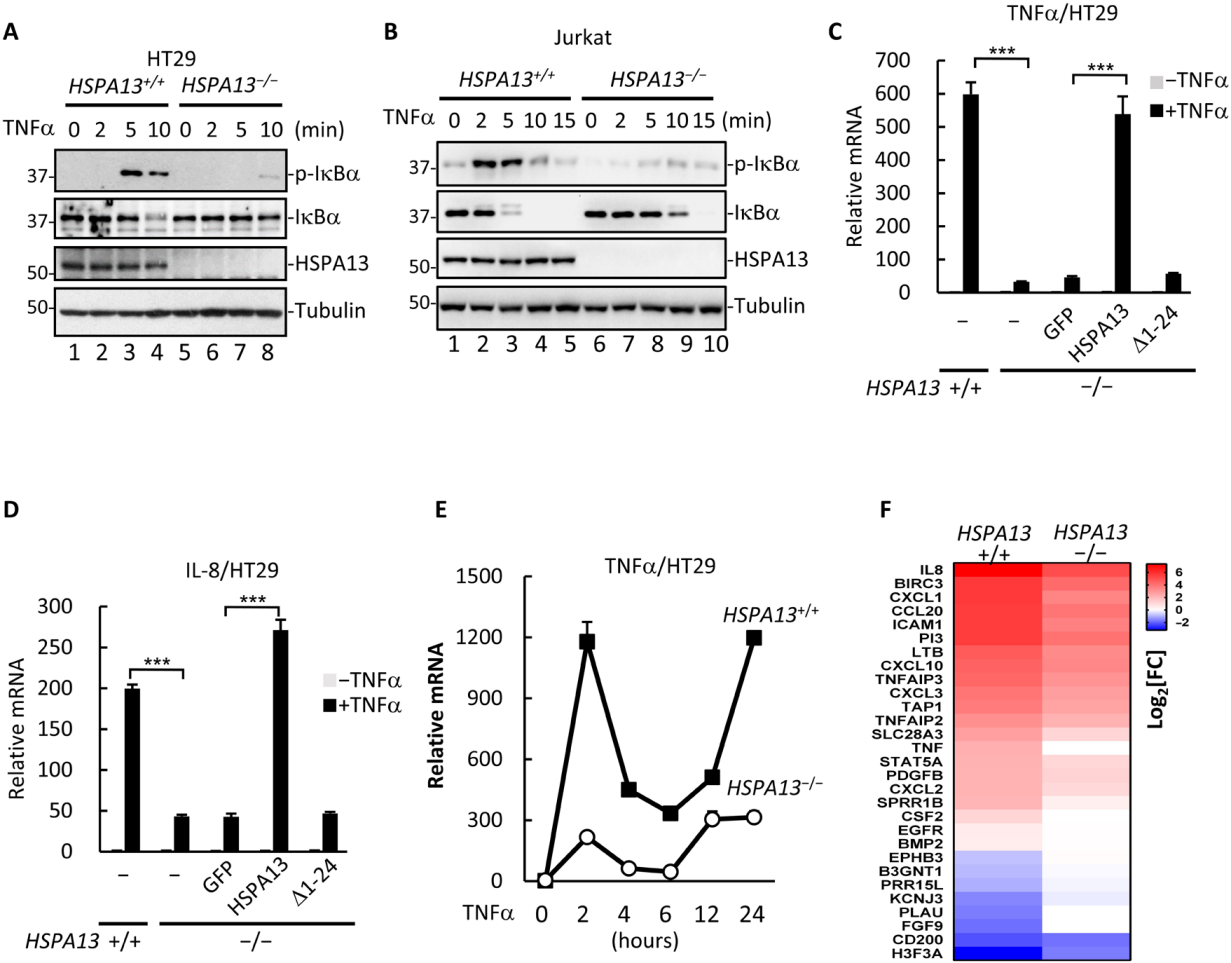
HSPA13 is required for TNFα-induced NF-κB signaling. (**A**) HT29 *HSPA13^+/+^* or *HSPA13*^−/−^ cells were treated with TNFα as indicated. Cell lysates were analyzed by Western blotting using the indicated antibody. (**B**) Jurkat *HSPA13*^−/−^ cell line was generated by using CRISPR-Cas9 system. Jurkat *HSPA13^+/+^* or *HSPA13*^−/−^ cells were treated with TNFα as indicated. Cell lysates were analyzed by Western blotting using the indicated antibody. (**C** and **D**) HT29 parental cells or *HSPA13*^−/−^ HT29 cells that stably expressed GFP, GFP-HSPA13, or GFP–Δ1-24 mutant were treated with TNFα for 4 hours. Total RNAs were extracted for qRT-PCR analysis to detect TNFα (C) and IL-8 (D) mRNA production. (**E**) HT29 *HSPA13^+/+^* or *HSPA13*^−/−^ cells were treated with TNFα for the indicated time. Total RNAs were extracted for qRT-PCR analysis. (**F**) HT29 *HSPA13^+/+^* or *HSPA13*^−/−^ cells were treated with TNFα for 4 hours. The heatmap showed fold changes of the selected genes in response to TNFα. All data shown are means (±SD) of three independent experiments. ***P < 0.001, by Student’s *t* test.

We next sought to investigate whether HSPA13 could control outcomes of TNFα signaling. As shown in Fig. 4A, loss of HSPA13 markedly suppressed IκBα phosphorylation and attenuated IκBα degradation in response to TNFα in HT29 cells (lanes 7 and 8). We then knocked out *HSPA13* in Jurkat cells using CRISPR-Cas9 system (fig. S3D). HSPA13 dependence of TNFα-induced IκBα phosphorylation was also observed in Jurkat cells (Fig. 4B, compare lanes 7 to 9 to lanes 2 to 4), suggesting a cell type–independent role of HSPA13 in NF-κB signaling. Consistently, stable expression of Flag-HSPA13 fully restored the TNFα-induced IκBα phosphorylation in *HSPA13*^−/−^ HT29 cells (fig. S5A).

Accordingly, TNFα-induced transcription of TNFα and interleukin-8 (IL-8) was markedly suppressed in *HSPA13*^−/−^ cells (Fig. 4, C and D). This defect in NF-κB–mediated transcriptional activation was largely rescued by reintroduction of HSPA13. By contrast, the Δ1-24 mutant failed to restore NF-κB signaling in *HSPA13*^−/−^ cells. Remarkably, attenuation of NF-κB signaling in *HSPA13*^−/−^ cells was also observed in long-term TNF-α treatment, as the second wave of TNFα mRNA up-regulation was severely weakened in *HSPA13*^−/−^ cells than in wild-type cells (Fig. 4E). Furthermore, we performed RNA sequencing (RNA-seq) experiments and analyzed a group of known TNFα target genes, including TNFα, IL-8, BIRC3, CXCL1, CCL20, ICAM1, EPHB3, and FGF9 (*35*). As shown in Fig. 4F, TNFα induced up-regulation or repression of most of the selected target genes in parental HT29 cells, but not or to a lesser extent in the HSPA13-null cells, demonstrating that HSPA13 was required for maximum expression of target genes in response to TNFα in HT29 cells. These results suggest that HSPA13 is a substantive regulator heightening TNFα-induced NF-κB activation.

### HSPA13 protects cells against TNFα-induced apoptosis and necroptosis

**Fig. 5.**
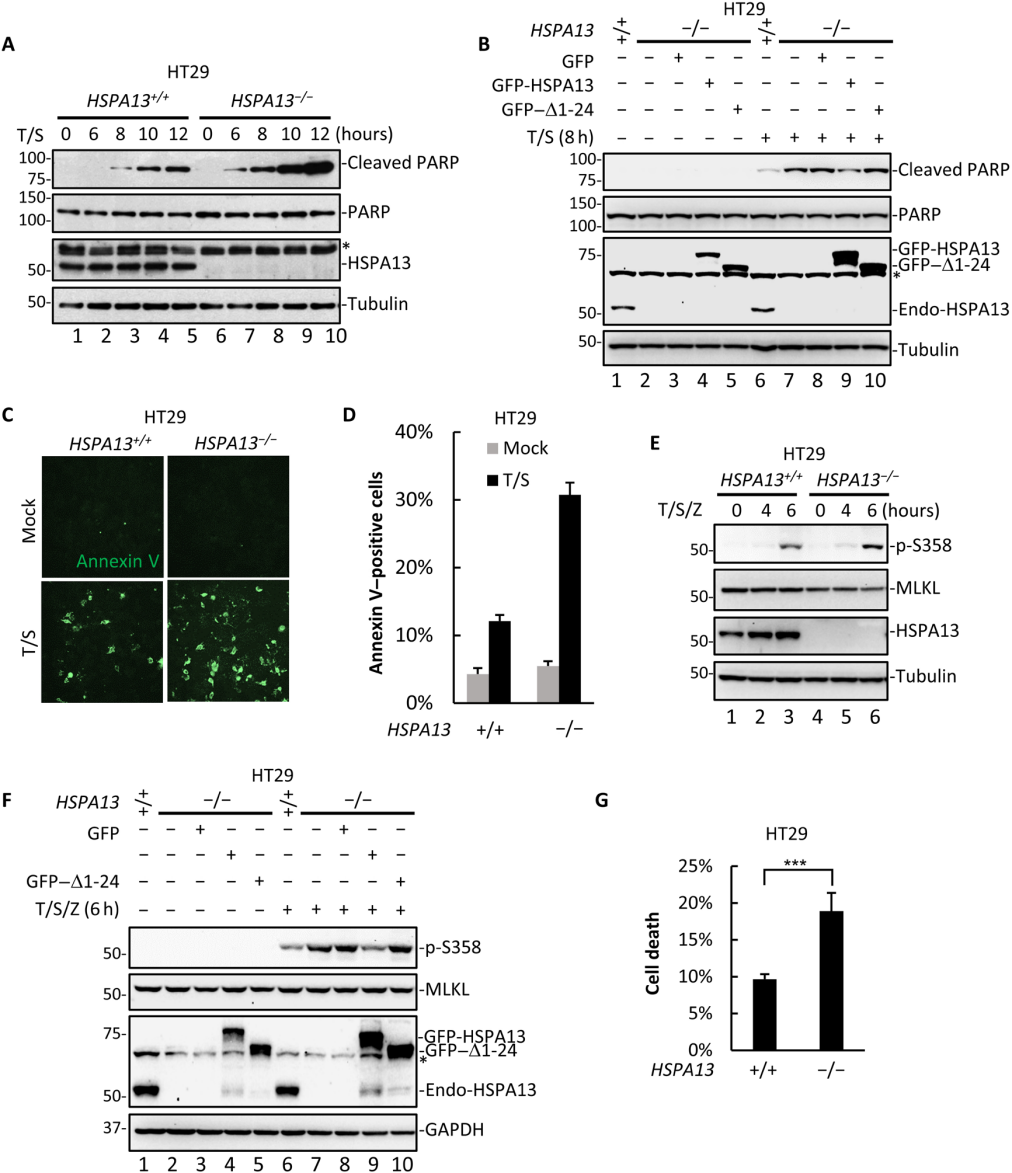
Loss of HSPA13 promotes TNFα-induced apoptosis and necroptosis. (**A**) HT29 *HSPA13^+/+^* or *HSPA13*^−/−^ cells were treated with T/S for the indicated time. Cell lysates were analyzed by Western blotting using the indicated antibody. (**B**) HT29 parental cells or *HSPA13*^−/−^ HT29 cells that stably expressed GFP, GFP-HSPA13, or GFP–Δ1-24 mutant were treated with T/S for 8 hours. Cell lysates were analyzed by Western blotting using the indicated antibody. (**C**) *HSPA13^+/+^* or *HSPA13*^−/−^ HT29 cells were treated with T/S. After 12 hours, cells were stained with annexin V. Images were taken with a fluorescence microscope. (**D**) Ratio of relative annexin V–positive cells in (C) was quantified by fluorescence-activated cell sorting (FACS). (**E**) *HSPA13^+/+^* or *HSPA13*^−/−^ HT29 cells were treated with T/S/Z for the indicated time. Cell lysates were analyzed by Western blotting using the indicated antibody. p-S358, MLKL-phospho-S358. (**F**) HT29 parental cells or *HSPA13*^−/−^ HT29 cells that stably expressed GFP, GFP-HSPA13, or GFP–Δ1-24 mutant were treated with T/S/Z for 6 hours. Cell lysates were analyzed by Western blotting using the indicated antibody. (**G**) *HSPA13^+/+^* or *HSPA13*^−/−^ HT29 cells were treated with T/S/Z for 8 hours. Cell viability was determined by measuring ATP levels using a CellTiter-Glo kit. All data shown are means (±SD) of three independent experiments. ***P < 0.001, by Student’s *t* test.

TNFα represents a double-edged sword as it activates inflammatory response to promote cell survival and also triggers cell demise via apoptosis and necroptosis. We then determined whether HSPA13 could regulate TNFα-induced cell death. We assessed T/S-induced RIP1-dependent apoptosis by measuring the level of cleaved poly(adenosine diphosphate–ribose) polymerase (PARP), a substrate of activated caspase 3. In agreement with the notion that HSPA13 deficiency accelerates the transition of complex I to complex IIa/IIb, it profoundly increased the level of T/S-induced PARP cleavage (Fig. 5A, lanes 8 to 10). T/S-induced PARP cleavage could be attenuated by reintroduction of wild-type HSPA13, but not the Δ1-24 mutant (Fig. 5B, compare lanes 9 and 10 to lane 8). Consistently, annexin V staining also showed that apoptotic cells were robustly increased in *HSPA13*^−/−^ cells (Fig. 5, C and D).

We also investigated whether HSPA13 deficiency altered TNFα-induced necroptosis. RIP3 induces phosphorylation of MLKL T357/S358 during necroptosis, which represents the key event in necroptotic execution (*22*, *36*). RIP3-mediated S358 phosphorylation of MLKL was induced upon T/S/Z treatment (6 hours) (Fig. 5E). This phosphorylation was profoundly increased in *HSPA13*^−/−^ cells (Fig. 5F, lanes 7 and 8), whereas reintroduction of stably expressed HSPA13 (lane 9), but not the Δ1-24 mutant (lane 10), largely reduced the level of S358 phosphorylation in *HSPA13*^−/−^ cells. Furthermore, necroptotic cell death was markedly increased in *HSPA13*^−/−^cells based on measurement of adenosine triphosphate (ATP) depletion level (Fig. 5G). Similar phenomenon was observed by staining necrotic cells with propidium iodide (PI) (fig. S5, C and D). Together, these results demonstrate that HSPA13 is a prosurvival regulator limiting the extent of TNFα-induced apoptosis and necroptosis.

### The regulatory function of HSPA13 depends on both TNFR1 and RIP1

**Fig. 6.**
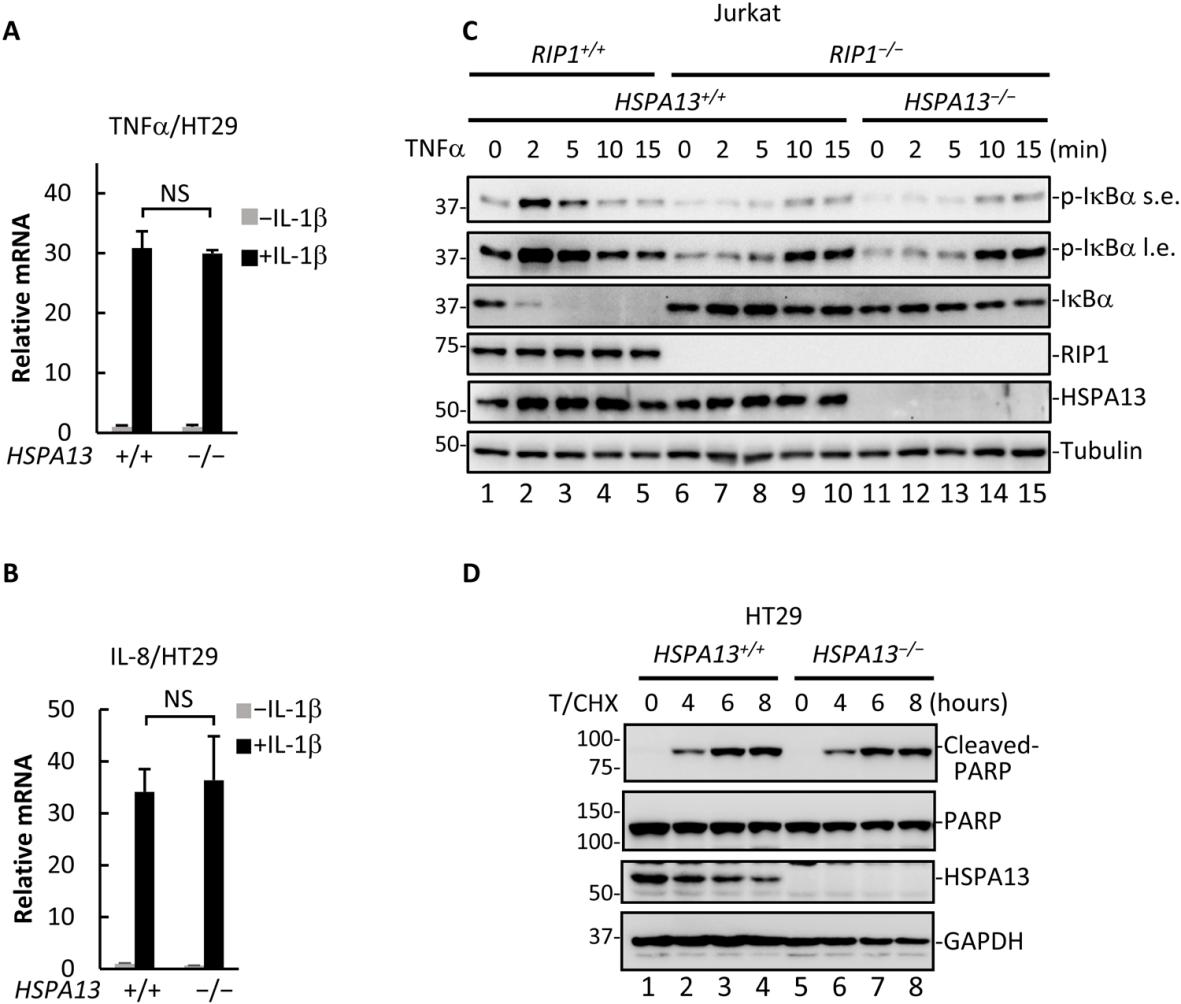
HSPA13 modulates outcomes of TNFα signaling in a RIP1- and TNFR1-dependent manner. (**A** and **B**) HT29 *HSPA13^+/+^* or *HSPA13*^−/−^ cells were treated with IL-1β (10 ng/ml) for 2 hours. Total RNAs were extracted for qRT-PCR analysis to detect TNFα (A) and IL-8 (B) mRNA production. (**C**) HSPA13 was knocked out in Jurkat *RIP1^−/−^* cells by using CRISPR-Cas9 system. Cells were treated with TNFα for the indicated time. Cell lysates were analyzed by Western blotting using the indicated antibody. s.e., short exposure; l.e., long exposure. (**D**) HT29 *HSPA13^+/+^* or *HSPA13*^−/−^ cells were treated with TNFα (20 ng/ml; T) along with CHX (10 μg/ml) for the indicated time. Cell lysates were analyzed by Western blotting using the indicated antibody. All data shown are means (±SD) of three independent experiments. NS (no significance), *P* > 0.05.

As noted earlier, HSPA13 modulates TNFα signaling by bridging the TNFR1-RIP1 interaction. We thus wondered whether regulation of NF-κB signaling by HSPA13 is restricted to the TNFR1 pathway. IL-1β binding of IL1R results in the activation of NF-κB signaling in both RIP1-dependent and RIP1-independent manner (*37*). It appeared that loss of HSPA13 did not attenuate IL-1β–induced transcriptional activation of TNFα (Fig. 6A) and IL-8 (Fig. 6B), which apparently differed from its effect on TNFα-induced signaling (Fig. 4).

Next, we examined RIP1-independent NF-κB activation in response to TNFα. To this end, we took advantage of *RIP1^−/−^* Jurkat cells that undergo weak NF-κB activation involving the linear ubiquitin chain assembly complex (LUBAC) in response to TNFα (Fig. 6C, lanes 9 and 10) (*38*, *39*). HSPA13 was further deleted using CRISPR-Cas9 strategy to generate *RIP1^−/−^/HSPA13^−/−^* Jurkat cells (fig. S3E). Intriguingly, HSPA13 was dispensable for NF-κB activation in *RIP1^−/−^* Jurkat cells (Fig. 6C, compare lanes 14 and 15 to lanes 9 and 10). Furthermore, combined treatment of TNFα and protein synthesis inhibitor cycloheximide (CHX) also triggers apoptosis, yet independently of RIP1 (*16*). As shown in Fig. 6D, this process was not influenced by ablation of *HSPA13*. Together, these data strongly suggest that HSPA13 governs outcomes of TNFα signaling in a TNFR1/RIP1-dependent manner.

### HSPA13 regulates TNFα signaling in vivo

**Fig. 7.**
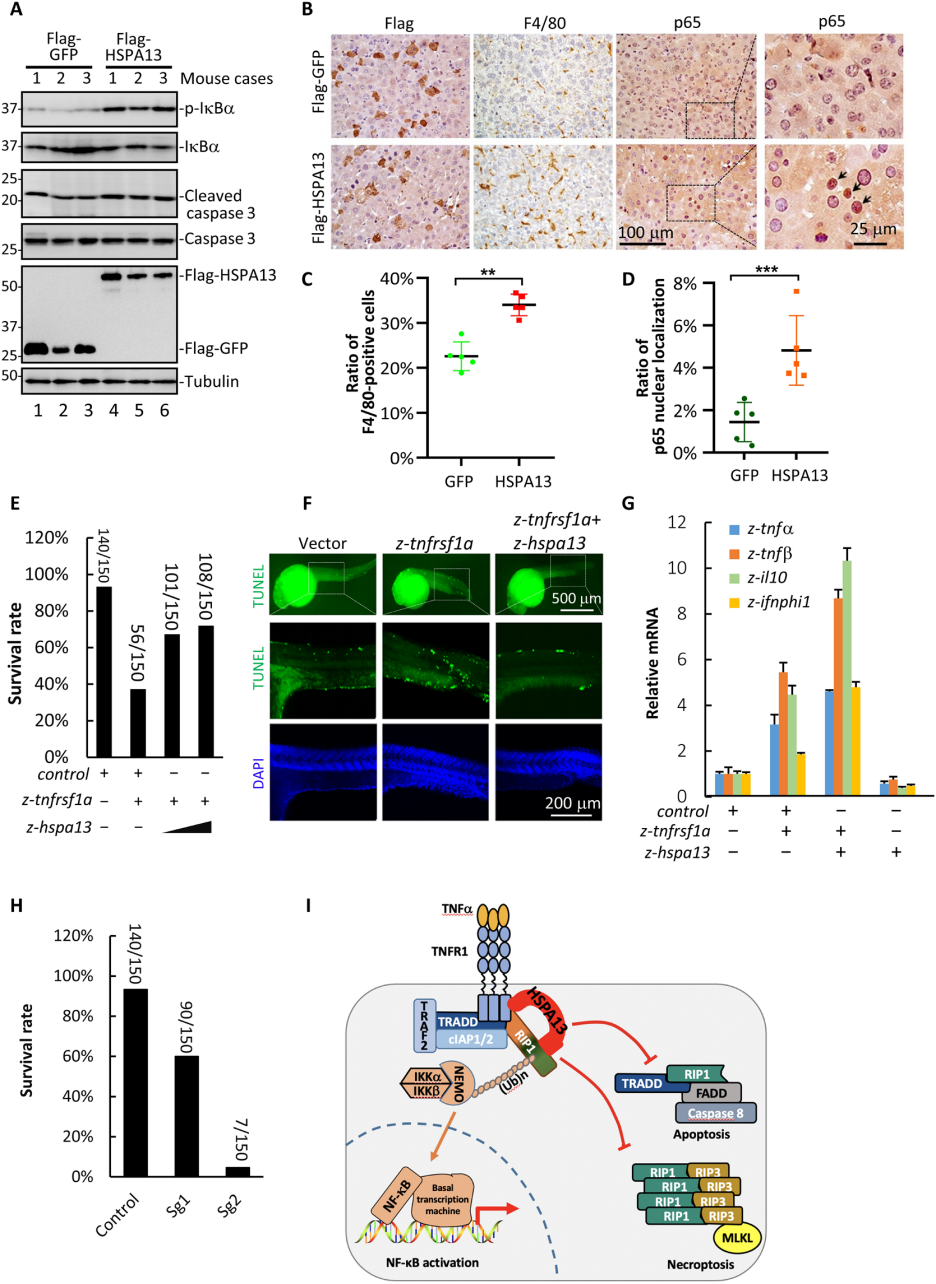
HSPA13 regulates TNFα signaling in vivo. (**A**) Four-week ICR mice were injected with Flag-GFP (*n* = 3) or Flag-HSPA13 (*n* = 3) via hydrodynamic injection system. Forty-eight hours after injection, lysates of mouse livers were analyzed by Western blotting. (**B**) Sections of livers were stained for Flag, macrophage marker F4/80, and p65. (**C** and **D**) Positive staining cells in (B) were counted using Image-Pro Plus software. F4/80-positive cells were normalized with hematoxylin staining nucleus (C). Cells with p65 nuclei localization were normalized with hematoxylin staining nucleus (D). (**E**) One-cell-stage zebrafish embryos were microinjected with 100 pg of zebrafish *tnfrsf1a* (*z-tnfrsf1a*) mRNA, 20 or 50 pg of *z-hspa13* mRNA, or control mRNA with a total amount of 150 pg. Embryonic survival rate was scored at 8 hpf. (**F**) At 24 hpf, survived zebrafish embryos in experiment (E) were fixed and subjected to TUNEL assay analysis. Images were taken under confocal microscopy. (**G**) At 24 hpf, survived zebrafish embryos in experiment (E) were subjected to qRT-PCR analysis to determine expression of *z-tnf*α, *z-tnf*β, *z-il-10*, and *z-ifnphi1*. (**H**) One-cell-stage zebrafish embryos were microinjected with Cas9 mRNA together with guide RNAs targeting *z-hspa13* (Sg1 or Sg2) or control guide RNA. At 24 hpf, embryonic survival rate was scored. (**I**) Working model. All data shown are means (±SD) of three independent experiments. ***P < 0.001 and **P < 0.01, by Student’s *t* test.

Because precise control of TNFα signaling is of paramount importance in maintaining tissue homeostasis, we investigate the role of HSPA13 in vivo. By using hydrodynamic injection of HSPA13 transgene in mouse hepatocytes (*40*), we achieved transient expression of HSPA13 in 10 to 20% of hepatocytes (fig. S6). In this approach, transient liver inflammation is commonly observed due to liver injury by high pressure. Notably, expression of HSPA13 increased the phosphorylation of IκBα, indicating a boost in inflammatory NF-κB activation (Fig. 7A, lanes 4 to 6). In contrast, there was little change on cleavage of procaspase 3 in HSPA13-expressing mouse liver tissues (Fig. 7A). Immunohistochemistry (IHC) analysis also showed that overexpression of HSPA13 enhanced translocalization of p65 from cytoplasm to nuclei and resulted in increased macrophage accumulation in mouse livers (Fig. 7, B to D). These results demonstrate that excess of HSPA13 promotes inflammatory response in mouse liver tissues.

Like mammals, zebrafish (*Danio rerio*) exhibits TNFα-mediated inflammatory and cell death responses (*41*, *42*). Sequence analysis shows that HSPA13 is highly conserved among vertebrates (fig. S7). Thus, we sought to examine the function of HSPA13 in zebrafish by challenging with ectopic expression of zebrafish TNFR1 homolog (*z-tnfrsf1a*). We found that *z-tnfrsf1a* induced severe death of embryos, and remarkably, coinjection of *z-hspa13* with *z-tnfrsf1a* resulted in a drastic reduction in embryonic death (Fig. 7E). Furthermore, *z-tnfrsf1a* produced notable morphological defects in embryos surviving beyond 24 hours postfertilization (hpf), which was also prevented by coinjection of *z-hspa13* (fig. S8, A and B). To examine whether *z-hspa13* affected apoptotic response upon *z-tnfrsf1a* challenge, we performed TUNEL (terminal deoxynucleotidyl transferase–mediated deoxyuridine triphosphate nick end labeling) assays in 24-hpf embryos and found that expression of *z-tnfrsf1a* profoundly increased TUNEL-positive cells (Fig. 7F). Consequently, coexpression of *z-hspa13* prevented *z-tnfrsf1a*–mediated apoptosis (Fig. 7F). Next, we characterized levels of inflammatory cytokines in zebrafish injected with *z-tnfrsf1a*. We found that *z-tnfrsf1a* induced high levels of *z-tnf*α, *z-tnf*β, *z-il-10*, and *z-ifnphi1*, which were amplified by *z-hspa13* (Fig. 7G). Moreover, we knocked down the *z-hspa13* gene using CRISPR-Cas9. Quantitative reverse transcription polymerase chain reaction (qRT-PCR) analysis indicated that ~30 and 80% efficiency of HSPA13 knockdown was achieved by two different guide RNAs, respectively (fig. S8, C and D). Similar to the results in cultured cells (Fig. 5), loss of *z-hspa13* in zebrafish induced extensive embryonic death, and the extent of death was well correlated to the knockdown efficiency (Fig. 7H). Knockdown of *z-hspa13* also caused severe morphological defects (fig. S8E) and massive apoptosis (fig. S8F). Collectively, these results demonstrate that *z-hspa13* also functions as a checkpoint for limiting the sensitivity to cytotoxicity of TNFα signaling but amplifying the inflammatory response.

## DISCUSSION

RIP1 has paradoxical functionality in orchestrating TNFα signaling to opposite consequences: cell survival or cell death (*4*). However, in most cell types, TNFα stimulation does not induce death but triggers canonical NF-κB–dependent induction of genes encoding prosurvival and proinflammatory molecules (*24*). *RIP1^−/−^* mice were born normally and died soon after birth (*43*), and this lethality is fully rescued by deletion of both *Caspase 8/FADD* and *RIP3* (*26*). Therefore, RIPK1 is considered to suppress programmed death pathways including caspase 8/FADD-mediated apoptosis and RIP3-driven necroptosis, and the activity of RIP1 should be precisely controlled in vivo. Recent studies have revealed that ubiquitination and phosphorylation of RIP1 determines the downstream output of RIP1 (*44*–*49*). In complex I, RIPK1 is modified with K63 and linear ubiquitination by cIAP1/2 and LUBAC, resulting in activation of NF-κB signaling and subsequent transcription-dependent suppression of cell death (*12*, *44*). Simultaneously, phosphorylation of RIP1 by complex I–recruited TAK1, IKK, and MK2 restricts cell death by inactivating RIP1 and blocking the formation of complex II (*45*–*47*). However, the mechanistic regulation of RIP1 recruitment to complex I remains poorly documented. In this study, we identify HSPA13 as a checkpoint to govern the distribution of RIP1 in diverse signaling complexes and modulate the cellular consequences in response to TNFα.

Our results provide the first evidence for the prosurvival function of HSPA13 in response to TNFα stimuli. The observation that TNFα treatment induces the recruitment of HSPA13 to complex I indicates that HSPA13 is an additional component of this membrane-associated complex. Because HSPA13 strongly interacts with both TNFR1 and RIP1 (Fig. 2), HSPA13 appears to serve as an additional bridging factor for stabilizing the TNFR1-RIP1 complex. HSPA13 deficiency suppresses RIP1 recruitment to the membrane-associated complex I and impairs subsequent K63 ubiquitination of RIP1, while it facilitates the RIP1 transition from complex I to the cytoplasmic death complex including complex IIa and complex IIb (Fig. 3). Thus, we propose a model in which HSPA13 functions as a link to keep RIP1 in complex I and as an effective barrier to limit the RIP1 death-promoting activity (Fig. 7I). Consistent with this model, HSPA13 deficiency markedly decreases NF-κB activation triggered by TNFα, while it facilitates cell death under apoptosis- or necroptosis-inducing conditions. Considering the expanding regulatory mechanisms for RIP1 activation, the HSPA13-RIP1 interaction may have other unrevealed functions in addition to stabilizing complex I. The observation that HSPA13 was coprecipitated modestly with cIAP1 using transiently expressed proteins (Fig. 2A) makes it more intriguing to investigate whether HSPA13 is involved in ubiquitination cascades in TNFα signaling and whether the ubiquitin “code,” in turn, affects HSPA13 recruitment.

We observed a marked consistency between alteration in the HSPA13 protein level and changes in signaling outputs and phenotypes both in mouse and in zebrafish models. Our identification of HSPA13 as a critical bridging factor for balancing the life or death decision induced by TNFα provides a new insight into further understanding the complexity of TNFα signaling networks. HSPA13 is reported to be increased in B220^+^ cells from patients with multiple myeloma or systemic lupus erythematosus, whereas mice with B cell–specific deletion of *HSPA13* have a reduction in plasmablasts, plasma cells, and antibody induction (*50*). Considering that TNFα signaling has a major role in B cell functions, it will be of immediate interest to explore the function of HSPA13 in B cell activation and development. Our finding also inspires further investigations to uncover the mechanistic link between HSPA13 and autoimmune diseases such as multiple myeloma or systemic lupus erythematosus.

## MATERIALS AND METHODS

### Reagents and antibodies

The following reagents were purchased commercially: TNFα (R&D Systems), IL-1β (R&D Systems), Z-VAD-FMK (Beyotime), and CHX (Sigma-Aldrich). Smac mimetic was a gift of X.-D. Wang (National Institute of Biological Sciences, Beijing, China). GST-TNFα was provided by Z.-P.X. (The First Affiliated Hospital, Zhengzhou University, Zhengzhou, Henan, China). GST-TUBE was a gift of J.J. (Zhejiang University, Hangzhou, Zhejiang, China).

All antibodies are commercially available (see table S1). Detailed information for all RT-PCR primers used is provided in table S2.

### Cell culture and transfection

HEK293T, HT29, and Jurkat cells were grown in RPMI 1640 with 10% fetal bovine serum. HEK293T cells were transfected with PEI (Polyscience). HSPA13 stable cells were obtained by using lentiviral infection. Briefly, lentiviral expression plasmids were transfected into HEK293T cells together with packaging plasmids (pMDL/pVSV-G/pRSV-Rev). Media containing viruses were collected after 48 hours and then used to infect host cells. Stable cells were then selected with puromycin (1 μg/ml; Sigma-Aldrich).

### Generation of knockout cell lines

Guide RNA sequence for HSPA13 (5′-ATTGTTCTGTTGGGGTG-3′) was subcloned into a CRISPR-Cas9–based vector modified from pX330 (*51*) with a puromycin resistance selection marker. This construct was transfected into HT29 and Jurkat cells. After selection in the presence of puromycin (1 μg/ml), single colonies were picked and verified by Western blotting analysis and DNA sequencing. Jurkat *RIP1^−/−^* cells were a gift of X. Lin (Tsinghua University, Beijing, China).

### Cell lysis, IP, and Western blotting analysis

Cells were harvested with IP lysis buffer [150 mM NaCl, 20 mM tris-HCl (pH 7.5), 0.5% Triton X-100, 0.5% NP-40, 5 mM glycero-2-phosphate, 10 mM NaF, 10% glycerol, 0.1% β-mercaptoethanol, and 5 mM EDTA; 5 mM N-ethylmaleimide (NEM), protease, and phosphatase inhibitors were added before use]. Cell lysates were then incubated together with protein A Sepharose CL-4B (GE Healthcare) and appropriate antibodies either overnight (for endogenous co-IP) or for 4 hours (for transiently expressed proteins) at 4°C. After several washes, immunoprecipitated proteins were eluted in SDS sample loading buffer, separated by SDS–polyacrylamide gel electrophoresis (PAGE), transferred onto polyvinylidene difluoride membranes (Millipore), and detected by Western blotting analysis.

### In vitro protein binding assay

GST or GST-fusion HSPA13 proteins were expressed in *Escherichia coli* strain DE3 and purified on glutathione-Sepharose 4B beads. In vitro translated RIP1 and TNFR1 were obtained by using the Quick Coupled Transcription/Translation System (Promega). In vitro binding was carried out through coincubation of GST-tagged HSPA13 and in vitro translated RIP1 for 2 hours in GST-binding buffer [150 mM NaCl, 50 mM tris-HCl (pH 7.5), and 0.5% NP-40; protease inhibitors were added before use] and analyzed by Western blotting.

### Pulldown of TNFR1-associated complex I

For pulldown of the ligand-receptor complex, cells were treated with GST-TNFα (1 μg/ml) for the indicated time (for *t* = 0, 200 ng of GST-TNFα was added at the time immediately after cell lysis). Cells were then lysed in IP lysis buffer. Cell lysates were incubated with glutathione-Sepharose 4B beads (GE Healthcare) overnight at 4°C. After several washes, retrieved proteins were analyzed by SDS-PAGE and Western blotting.

### TUBE assay

Following specified stimulation, cultured cells were lysed in TUBE lysis buffer [150 mM NaCl, 50 mM tris-HCl (pH 7.5), 0.5% NP-40, and 10 mM NaF; 5 mM NEM, protease, and phosphatase inhibitors were added before use]. Cell lysates were incubated with 6 μg of purified GST-TUBE (on glutathione-Sepharose) for 2 hours at 4°C. After several washes, retrieved proteins were analyzed by SDS-PAGE and Western blotting.

### Mass spectrometric analysis

HEK293T cells were transfected with plasmid encoding SFB-RIP1. After 24 hours, cell lysates were harvested by IP lysis buffer first incubated with streptavidin Sepharose (GE Healthcare) for 4 hours at 4°C, and after several washes, streptavidin Sepharose–bound proteins were eluted with biotin (1 mg/ml). Then, the eluent was incubated with S-protein agarose beads (Novagen) overnight at 4°C. After being washed for three times, immobilized proteins were eluted in SDS sample loading buffer and separated by SDS-PAGE. The protein mixtures were analyzed by nano–liquid chromatography–tandem mass spectrometry analysis for protein identification at Phoenix National Proteomics Core service as described (*52*).

### Proximity ligation assay

HeLa cells grown on coverslips were fixed with 4% formaldehyde for 20 min and then incubated with 0.5% Triton X-100 for 20 min. The PLA was performed using the Duolink In Situ Red Starter Kit (Sigma-Aldrich). Florescence images were acquired with a Zeiss LSM710 confocal microscope (Carl Zeiss).

### RNA-seq data analysis

RNA-seq data analysis was performed as previously described (*53*). Briefly, sequencing reads were trimmed to 50 base pairs (bp) and mapped to the human transcriptome (UCSC hg19). Gene expression was quantified by normalized fragments per kilobase of exon per million mapped fragments (FPKM). Genes with FPKM less than 1 in all samples were considered not expressed and therefore excluded in the subsequent analysis. For the remaining genes, all FPKM values less than 1 were set to 1. Gene expression change upon TNFα stimulation was calculated as the ratio of FPKM values.

### Cell survival assay

Cells were treated as indicated in the figure legends. For apoptosis quantification, cells were collected and stained with annexin V using an Annexin V-FITC/PI apoptosis kit (Liankebio). Annexin V–positive cells were analyzed using FACSCalibur (BD Biosciences). For necroptosis quantification, cell survival assay was performed using the CellTiter-Glo Luminescent Cell Viability Assay Kit (Promega).

### Hydrodynamic tail vein injection

Ectopic gene expression in mouse liver was carried out by hydrodynamic tail vein injection as described previously (*40*). Briefly, 50 μg of mammalian expression plasmids was mixed with Ringer’s solution in a volume equal to 10% of the body weight. The mixture was then injected into the tail vein of 4-week-old female ICR mouse in 5 to 7 s. After 48 hours, blood was obtained by cardiac puncture and livers were collected for Western blotting and IHC analysis. All animal studies were approved by the Zhejiang University Committee for Experimental Animal Studies and Ethics.

### Immunohistochemistry

Mouse livers were fixed, paraffin-embedded, and sliced. Sections were deparaffinized through xylene and graded ethanol solutions. After antigen retrieval, sections were incubated with primary antibodies for 1 hour and biotin-labeled secondary antibodies for 30 min at room temperature. Biotin signal was detected by a Vectastain ABC kit and a DAB peroxidase substrate kit (Vector Laboratories), and cell nuclei were stained with hematoxylin.

### Zebrafish embryo assay

Ectopic expression of exogenous genes in AB wild-type zebrafish embryos was performed as previously described (*54*). Briefly, in vitro transcribed mRNA by the mMESSAGE mMACHINE SP6 Transcription Kit (Life Technologies) was injected into yolk of one-cell-stage embryos. Embryos were then raised at 28.5°C. The survival rate was counted at 8 and 24 hpf, respectively. To monitor transcription of inflammatory cytokines, survived embryos at 24 hpf were collected for RNA extraction and qRT-PCR analysis. To detect apoptotic cells, survived embryos at 24 hpf were fixed and assayed by TUNEL using the ApopTag Fluorescein Direct In Situ Apoptosis Detection Kit (Millipore).

Detailed information of materials is available in the Supplementary Materials.

## Notes

View/request a protocol for this paper from *Bio-protocol*.
