# Supplementary material for: HSPA13 facilitates NF-κB–mediated transcription and attenuates cell death responses in TNFα signaling
Source: Sci Adv. 2021 Oct 6;7(41):eabh1756. doi: 10.1126/sciadv.abh1756 (PMC8494447; doi:10.1126/sciadv.abh1756)
Supplement: Supplementary file 1 — Supplementary Text Tables S1 and S2 Figs. S1 to S8 [file sciadv.abh1756_sm.pdf]

Science Advances
AAAS

Supplementary Materials for

HSPA13 facilitates NF-κB–mediated transcription and attenuates cell death responses in TNFα signaling

Chun Gao, Jianhua Deng, Hanchenxi Zhang, Xinran Li, Shuchen Gu, Mingjie Zheng, Mei Tang, Yezhang Zhu, Xin Lin, Jianping Jin, Long Zhang, Jun Huang, Jian Zou, Zong-Ping Xia, Ping-Long Xu, Li Shen, Bin Zhao, Xin-Hua Feng*

*Corresponding author. Email: fenglab@zju.edu.cn

Published 6 October 2021, *Sci. Adv.* **7**, eabh1756 (2021)
DOI: 10.1126/sciadv.abh1756

This PDF file includes:

Supplementary Text
Tables S1 and S2
Figs. S1 to S8

Supplementary information text

Materials and Methods

Plasmids

DNA fragments encoding human TNFR1, TRADD, TFAF2, CYLD, cIAP1, cIAP2, Caspase8, c-FLIP-L and FADD were gifts of Z. Xia (Zhengzhou University, Henan, China). The coding regions for human HSPA13, RIP1, RIP3 and MLKL were individually amplified by PCR from the human ORFeome collection (v.5.1). The coding regions for Zebrafish *z-hspa13* and *z-tnfrsf1a* were amplified by RT-PCR from total RNAs isolated from 24 hpf zebrafish embryos. The ORF of each individual gene was subcloned into pXF6F (N-terminal Flag tag), pXF3HM (N-terminal His and Myc tag), pXF4H (N-terminal HA tag), pXF1EG (N-terminal EGFP tag) or pRK5F vector (C-terminal Flag tag) for mammalian expression. HSPA13 ORF was subcloned into pGEX-6P-2 for *E. coli* expression. HSPA13 deletion mutants were generated by PCR-directed site mutagenesis. Lentiviral expression plasmids for Flag-HSPA13 and GFP-HSPA13 were constructed by subcloning HSPA13 ORF into pBOBi vector. The integrity of all expression plasmids was verified by DNA sequencing.

Subcellular fractionation

The fractionation assay was carried out utilizing Triton X-114 which undergoes phase transition at different temperatures as described(36). Briefly, HEK293T cells were lysed by Triton X−114 lysis buffer (150 mM NaCl, 20 mM HEPES [pH7.4], 2% Triton X−114; protease inhibitors added before use). Cell lysates were warmed at 30°C for 3 min and subsequently centrifuged at 1,500 × g for 5 min at room temperature. The detergent soluble fraction was separated into aqueous phase (Aq) and detergent phase (Det). The Aq phase was re-centrifuged to remove detergent contamination, and the Det phase was washed by basal buffer (150 mM NaCl, 20 mM HEPES [pH7.4]) and diluted to the same volume of the Aq phase. Samples were analyzed by SDS-PAGE and western blot

Supplementary Table 1. List of antibodies used in this study.

Antibodies	Suppliers (catalog number)
HSPA13	Proteintech Group (2667-2-AP)
Flag	Sigma-Aldrich (F3165)
GAPDH	Sigma-Aldrich (G8795)
γ-Tubulin	Sigma-Aldrich (T5326)
GFP	Santa Cruz Biotechnology (sc-9996)
Myc	Cell Signaling Technology (2276)
HA	Cell Signaling Technology (3724BC)
GST	Cell Signaling Technology (2624)

RIP1	Cell Signaling Technology (3493)
RIP3	Cell Signaling Technology (13526)
TNFR1	Cell Signaling Technology (3736)
IκBα	Cell Signaling Technology (4812)
p- IκBα	Abcam (ab92700)
P100/P52	Cell Signaling Technology (3017
MLKL-p-358	Abcam (ab187091)
MLKL	Millipore (MABC604)
PARP	Cell Signaling Technology (9632)
cleaved PARP	Cell Signaling Technology (5625)
Caspase 3	Cell Signaling Technology (9665)
Cleaved Caspase 3	Cell Signaling Technology (9664)
Caspase 8	Proteintech Group (13423-1-AP)
P65	Santa Cruz Biotechnology (sc-372)
F4/80	Bio-Rad (MCA497G)
HRP Goat anti Rabbit IgG	Jackson Immuno Research
HRP Rabbit anti Mouse IgG	Jackson Immuno Research

Supplementary Table 2. List of RT-PCR primers used in this study.

Primers	Sequence 5'-3'	Species
hGAPDH-Forward	CGACCACTTTGTCAAGCTCA	Human
hGAPDH-Reverse	TTACTCCTTGGAGGCCATGT	
hTNFα-Forward	CCCAGGGACCTCTCTCTAATCA	Human
hTNFα-Reverse	GCTTGAGGGTTTGCTACAACATG	
hIL-8-Forward	ATAAAGACATACTCCAAACCTTTCCAC	Human
hIL-8-Reverse	AAGCTTTACAATAATTTCTGTGTTGGC	
z-actin-Forward	ATGGATGAGGAAATCGCTC	Zebrafish
z-actin-Reverse	ATGCCAACCATCACTACTCCCTG	
z-tnfα-Forward	GCTGGATCTTCAAAGTCGGGTGTA	Zebrafish
z-tnfα-Reverse	TGTGAGTCTCAGCACACTTCCATC	
z-tnfβ-Forward	TGGATATGGAAGACACTCGC	Zebrafish
z-tnfβ-Reverse	GACCTTCTTCGTTTGGCTTC	
z-il-10-Forward	TTGTGGAGGGCTTTCCTTTA	Zebrafish
z-il-10-Reverse	ACGTGACATCCATAGGGACT	
z-ifn-Forward	TCTCTGAACCTGCTCAAGAA	Zebrafish
z-ifn-Reverse	TCCTCCACCTTTGACTTGTC	
z-hspa13-01-Forward	ATGGCCGGAGAAATGTCAAT	Zebrafish
z-hspa13-01-Reverse	ACTCCCACAGAACAGAAAG	
z-hspa13-02-Forward	TTGGGCTGGATCTTGGGAC	Zebrafish
z-hspa13-02-Reverse	TTCCTGGCCCTCATGTCCA	

Supplementary Figure 1

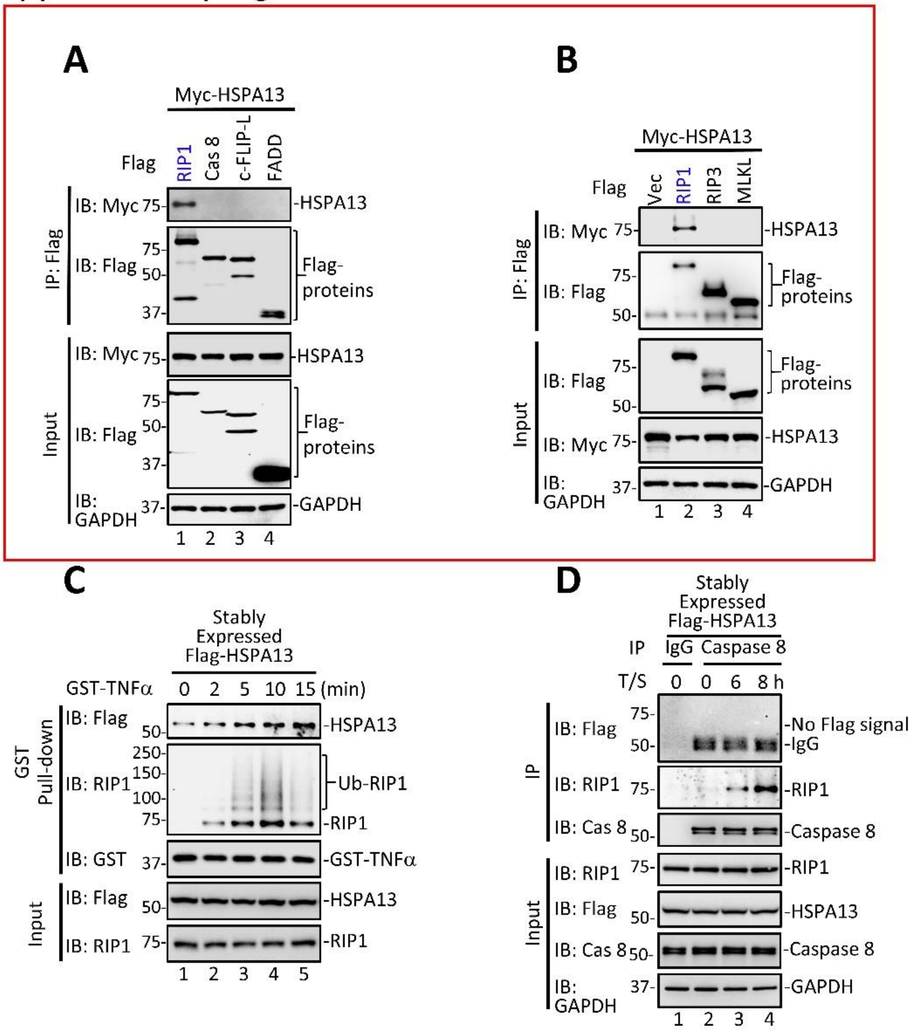

HSPA13 is a novel component of TNFR1-associated complex I. (A-B) HEK293T cells were transfected with indicated plasmids. After 24 h, Co-IP was carried out with anti-Flag. (C) HT29 cells stably expressing Flag-HSPA13 were stimulated with GST-TNFα (1 μg/ml) for indicated time. Complex I was purified using glutathione-sepharose and analyzed by western blotting. (D) HT29 cells stably expressing Flag-HSPA13 were stimulated with T/S for indicated time. Cell lysates were immunoprecipitated with anti-Caspase 8 antibody or control IgG antibody.

Supplementary Figure 2

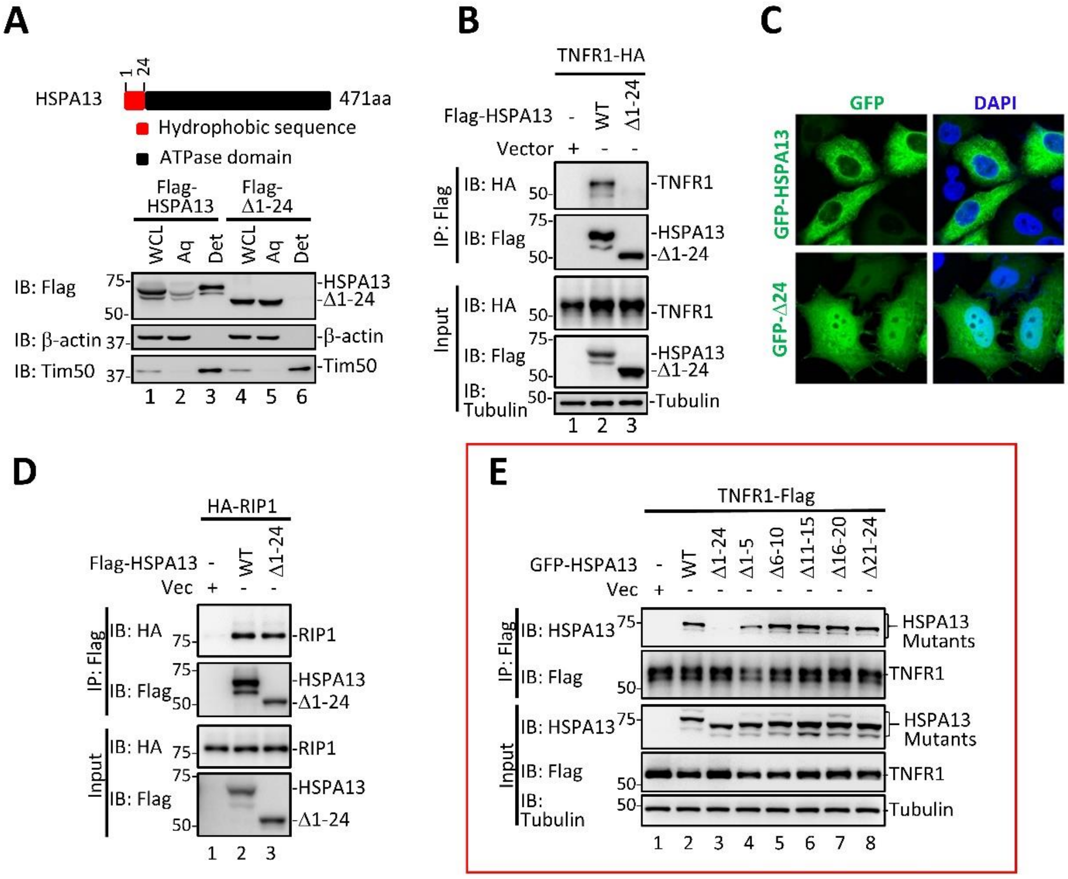

The N-terminal of HSPA13 is necessary for its binding to TNFR1. .(A) HEK293T cells were transfected as indicated. After 24 h, the cells were harvested and then separated into aqueous phase (Aq) and detergent phase (Det) .The samples were analyzed by western blot. β-actin and mitochondria membrane protein TIM50 were shown as loading controls for soluble protein and membrane protein, respectively. WCL, whole cell lysates. (B, D and E) HEK293T cells were transfected with indicated plasmids. After 24 h, Co-IP was carried out with anti-Flag. (C) Hela cells stably expressing GFP-HSPA13 or GFP-Δ1-24 mutant were fixed, and stained with DAPI (blue). Images were taken by confocal microscopy.

Supplementary Figure 3

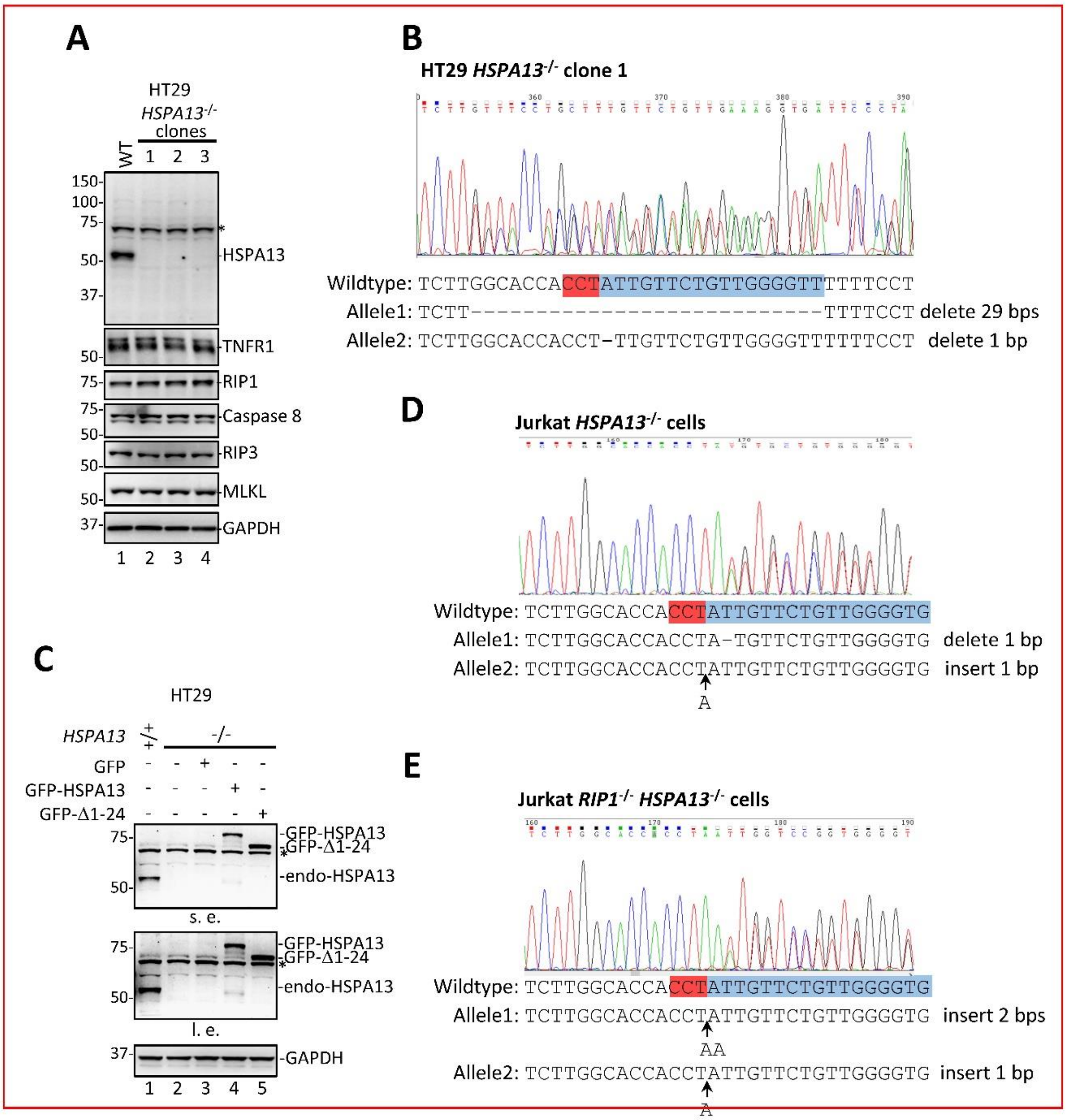

Knockout of *HSPA13* using CRISR/Cas 9 system. (A-B and D-E) HSPA13$^{-/-}$cell lines were generated by using CRISPR/Cas9 system. (A) The HT29 HSPA13$^{-/-}$clones were picked and analyzed by western blotting. (B and D-E) DNA sequencing analysis confirmed nucleotides insertion or deletion in CRISPR/Cas 9-edited cells. The sgRNA sequence (5'-ATTGTTCTGTTGGGGTG -3') is labeled in blue font and PAM recognition sequence highlighted in red. (C) GFP-HSPA13, GFP-Δ1-24 or GFP was stably expressed in HT29 *HSPA13*$^{-/-}$cells. Cell lysates were analyzed by western blotting. s.e., short exposure. l.e., long exposure. Endo-HSPA13, endogenous HSPA13.

Supplementary Figure 4

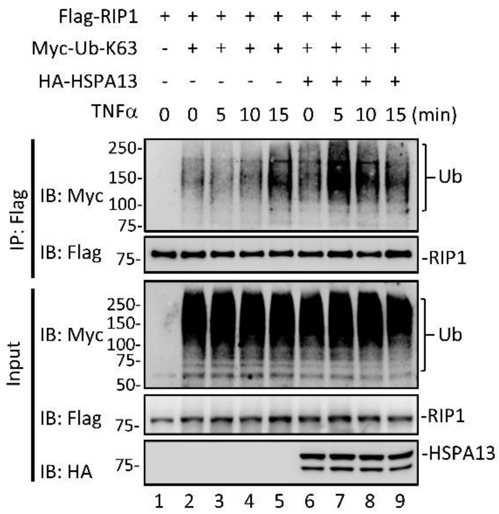

HSPA13 enhances K63-ubiquitination of RIP1. Myc-Ubiquitin mutant containing only one lysine at position 63 (Ub-K63), Flag-RIP1 and HA-HSPA13 were co-transfected to HEK293T cells as indicated. After 24 h, cells were treated with TNFα for indicated time. Immunoprecipitates and input were analyzed by western blotting.

Supplementary Figure 5

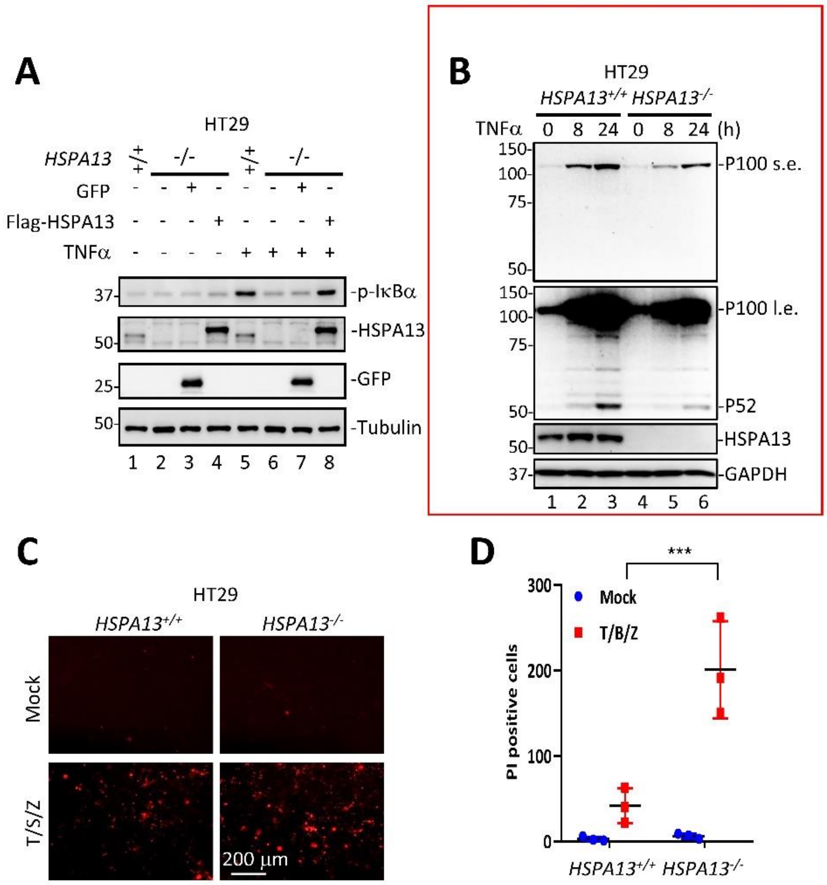

HSPA13 modulates outcomes of TNFα signaling. (A-B) Cells were treated as indicated and samples were analyzed by western blotting using indicated antibody. (C) *HSPA13*$^{+/+}$ or *HSPA13*$^{-/-}$ HT29 cells were treated with T/S/Z for 8 h. Necrotic cells were stained with PI. (D) Quantitation of necroptotic cell death in Panel B. Values are mean (± SD) of three independent experiments. ****P* < 0.001.

Supplementary Figure 6

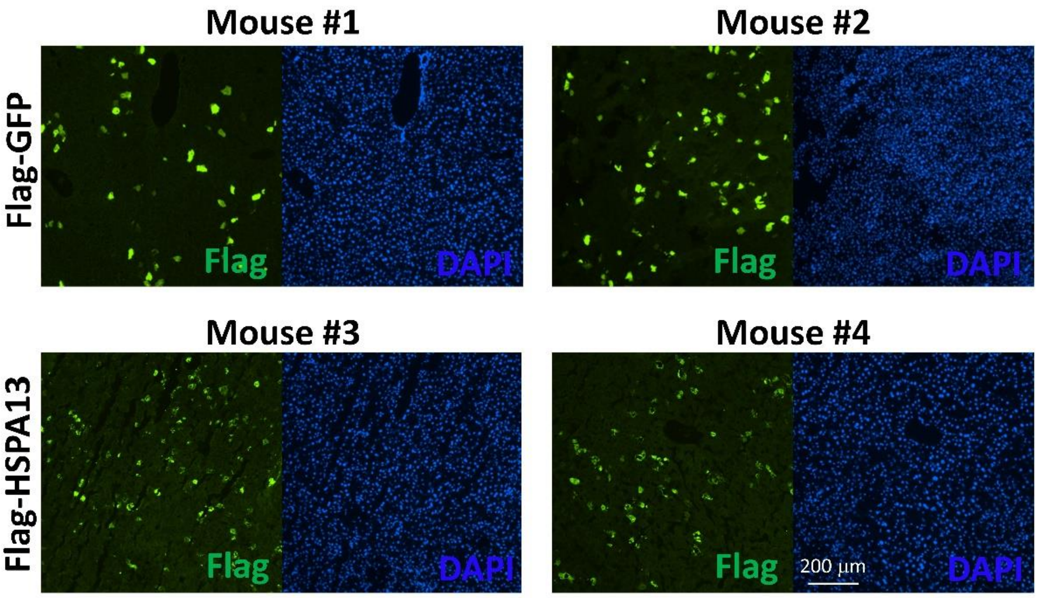

Ectopic expression via HDI system. Sections of livers in (Fig. 7A) were stained for Flag or DAPI. Images are representative of two mice .

Supplementary Figure 7

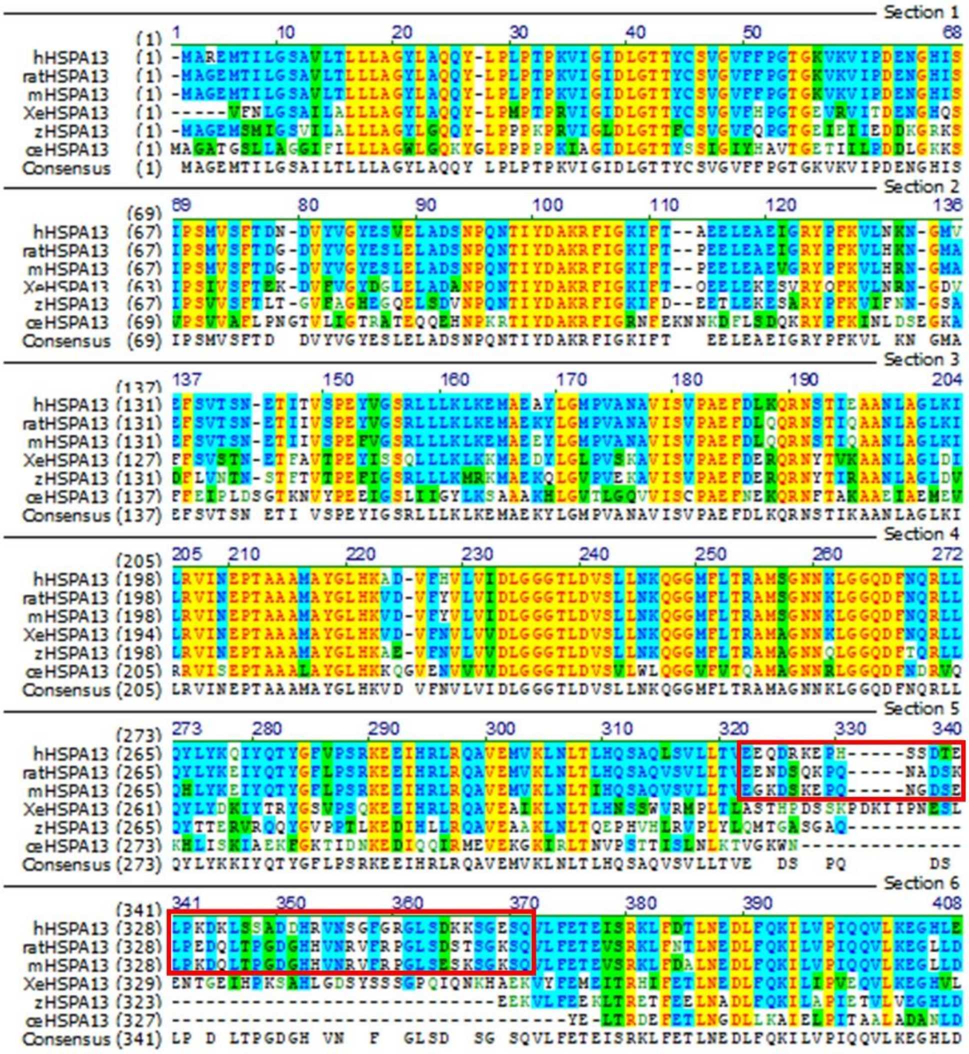

Amino-acid alignment of human HSPA13 (hHSPA13), Rat HSPA13, mouse HSPA13 (mHSPA13), zebrafish HSPA13 (zHSPA13), *Xenopus laevis* HSPA13 (XeHSPA13) and Caenorhabditis elegans *HSPA13 (CeHSPA13).* The unique mammalian-specific insertion (human HSPA13 aa314-358) was boxed in red.

Supplementary Figure 8

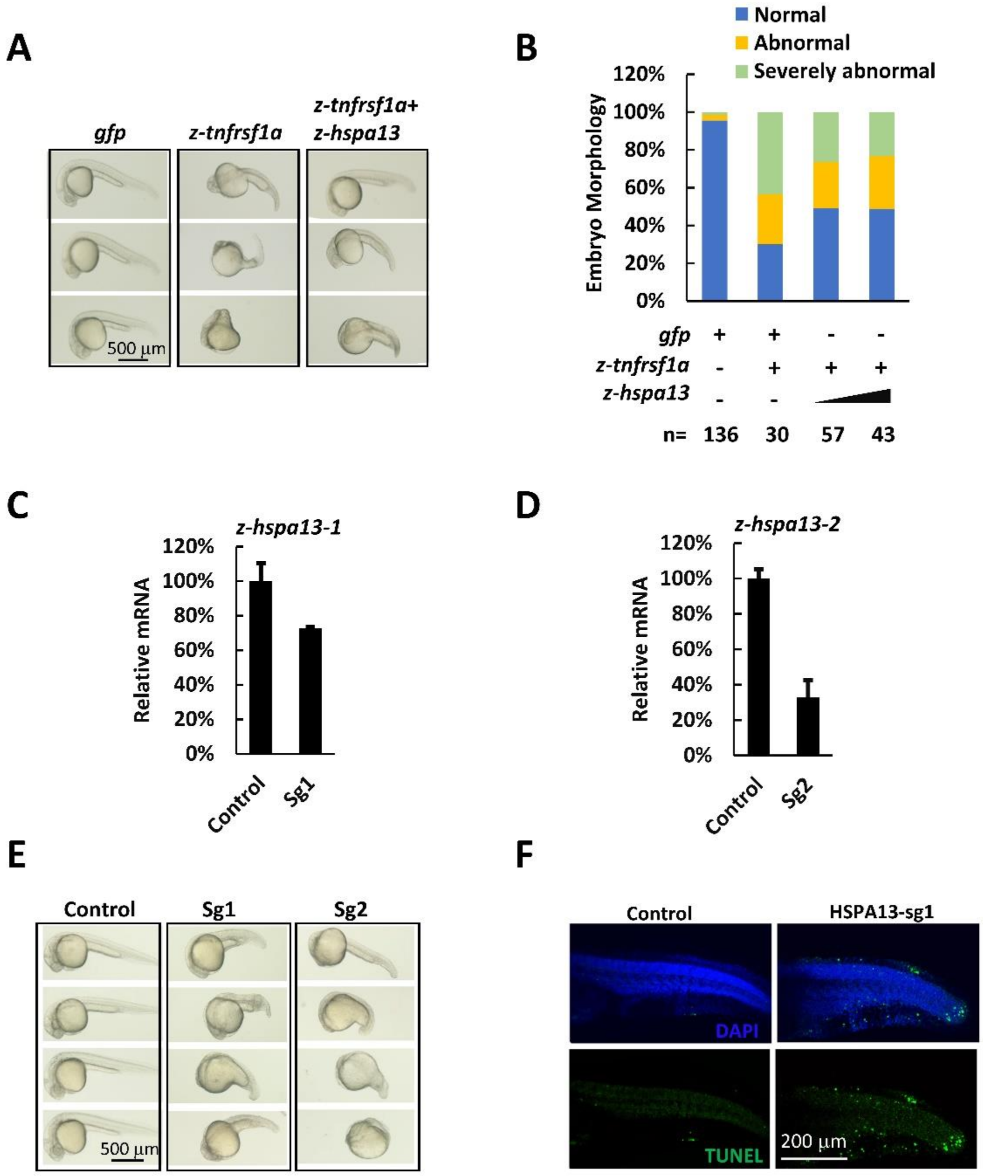

HSPA13 regulates TNFα signaling in zebrafish. (A-B) Survived zebrafish embryos in experiment (Fig 7E) were photographed at 24 hpf (A), and statistical data for phenotypes are shown in (B). (C-D) One-cell-stage zebfafish embryos were microinjected with Cas9 mRNA together with guide RNA target *z-hspa13* (Sg1 or Sg2) or control guide RNA. The reverse primers of *z-hspa13* were designed to overhang the PAM motif targeted by Sg1 or Sg2, respectively. At 24 hpf, total RNA was extracted for qRT-PCR analysis. Values are mean (± SD) of three independent experiments. (E) Survived zebrafish embryos in experiment (Fig 7G) were photographed at 24 hpf. (F) survived zebrafish embryos in experiment (Fig 7G) were fixed and subjected to TUNEL assay analysis. Images were taken under confocal microscopy.
